# The Experimental Oxime K027—A Promising Protector From Organophosphate Pesticide Poisoning. A Review Comparing K027, K048, Pralidoxime, and Obidoxime

**DOI:** 10.3389/fnins.2019.00427

**Published:** 2019-05-22

**Authors:** Dietrich E. Lorke, Georg A. Petroianu

**Affiliations:** ^1^Department of Cellular Biology and Pharmacology, Herbert Wertheim College of Medicine, Florida International University, Miami, FL, United States; ^2^Department of Anatomy and Cellular Biology, College of Medicine and Health Sciences, Khalifa University, Abu Dhabi, United Arab Emirates

**Keywords:** carbamates, cholinesterase, Cox analysis, obidoxime, paraoxon, pralidoxime, pyridostigmine, prophylaxis

## Abstract

Poisoning with organophosphorus compounds (OPCs) is a major problem worldwide. Standard therapy with atropine and established oxime-type enzyme reactivators (pralidoxime, obidoxime) is unsatisfactory. In search of more efficacious broad-spectrum oximes, new bispyridinium (K-) oximes have been synthesized, with K027 being among the most promising. This review summarizes pharmacokinetic characteristics of K027, its toxicity and *in vivo* efficacy to protect from OPC toxicity and compares this oxime with another experimental bisquaternary asymmetric pyridinium aldoxime (K048) and two established oximes (pralidoxime, obidoxime). After intramuscular (i.m.) injection, K027 reaches maximum plasma concentration within ∼30 min; only ∼2% enter the brain. Its intrinsic cholinesterase inhibitory activity is low, making it relatively non-toxic. *In vitro* reactivation potency is high for ethyl-paraoxon-, methyl-paraoxon-, dichlorvos-, diisopropylfluorophosphate (DFP)- and tabun-inhibited cholinesterase. When administered *in vivo* after exposure to the same OPCs, K027 is comparable or more efficacious than pralidoxime and obidoxime. When given as a pretreatment before exposure to ethyl-paraoxon, methyl-paraoxon, DFP, or azinphos-methyl, it is superior to the Food and Drug Administration-approved compound pyridostigmine and comparable to physostigmine, which because of its entry into the brain may cause unwanted behavioral effects. Because of its low toxicity, K027 can be given in high dosages, making it a very efficacious oxime not only for postexposure treatment but also for prophylactic administration, especially when brain penetration is undesirable.

## Introduction

Fatalities due to poisoning with organophosphorus compounds (OPCs) represent a major problem worldwide. OPCs are used for a broad range of purposes, ranging from parasiticides, for example, parathion, malathion, methyl-parathion, azinphos-methyl, terbufos, dichlorvos, or dicrotophos, to flame retardants, hydraulic liquids, and additives to lubricants (Gupta, 2006a). Because they are so extensively used, readily available, and also easy to synthesize, they are among the most frequent causes of accidental, professional, and suicidal intoxications. It is estimated that the number of fatalities per year reaches 200,000, with developing countries being the most affected (Eddleston et al., 2008; Chowdhary et al., 2014; King and Aaron, 2015). Moreover, they have also been abused in malicious poisonings, terrorist attacks, or chemical warfare (Antonijevic and Stojiljkovic, 2007; Petroianu, 2014; Myhrer and Aas, 2016; Masson and Nachon, 2017). The main toxic mechanism of OPCs is phosphylation (denoting phosphorylation or phosphonylation) of acetylcholinesterase (AChE), the enzyme responsible for hydrolyzing the neurotransmitter acetylcholine (ACh) at cholinergic synapses. ACh is the neurotransmitter at the neuromuscular junction of the somatic nervous system, at sympathetic and parasympathetic ganglia of the autonomic nervous system, at parasympathetic nerve terminals supplying smooth muscle, cardiac muscle, and glands, and at synapses in the central nervous system. AChE inhibition results in the accumulation of ACh at these cholinergic synapses and long-lasting stimulation of nicotinic and muscarinic ACh receptors. Muscarinic signs and symptoms can be memorized by the mnemonic DUMBBELLS (diarrhea, urination, miosis, bronchorrhea, bronchospasms, emesis, lacrimation, laxation, sweating); nicotinic stimulation leads to tachycardia, high blood pressure, muscle fasciculations, and, in severe cases, paralysis of respiratory muscles. Central nervous system symptoms are restlessness, seizures, and coma (Eyer et al., 2003; Balali-Mood and Balali-Mood, 2008). Death generally occurs due to respiratory insufficiency, generalized seizures, and/or multiorgan failure (Petroianu et al., 1998; Petroianu, 2005; Antonijevic and Stojiljkovic, 2007; Balali-Mood and Balali-Mood, 2008; Hrabetz et al., 2013).

Standard treatment combines, in addition to supportive therapy, three therapeutic approaches: blocking muscarinic stimulation by atropine, dephosphorylating inhibited AChE by oxime-type reactivators, and controlling seizures by benzodiazepines, for example, diazepam or midazolam. The oxime of choice in Japan, Great Britain, the United States, and France is pralidoxime; in the Netherlands, Finland, Norway, and Germany, obidoxime is used; HI-6 is standard in Sweden and Canada; and trimedoxime (TMB-4) is currently stored in some East European countries (Stojiljkovic and Jokanovic, 2006; Worek and Thiermann, 2013). However, several studies cast doubt on the efficacy of these oximes, particularly in the treatment of pesticide ingestion (Johnson et al., 2000; Eyer, 2007; Eddleston et al., 2008; Buckley et al., 2011; Blumenberg et al., 2018). Therefore, several research groups are working on the synthesis of more efficacious broad-spectrum oxime-type reactivators.

**FIGURE 1:**
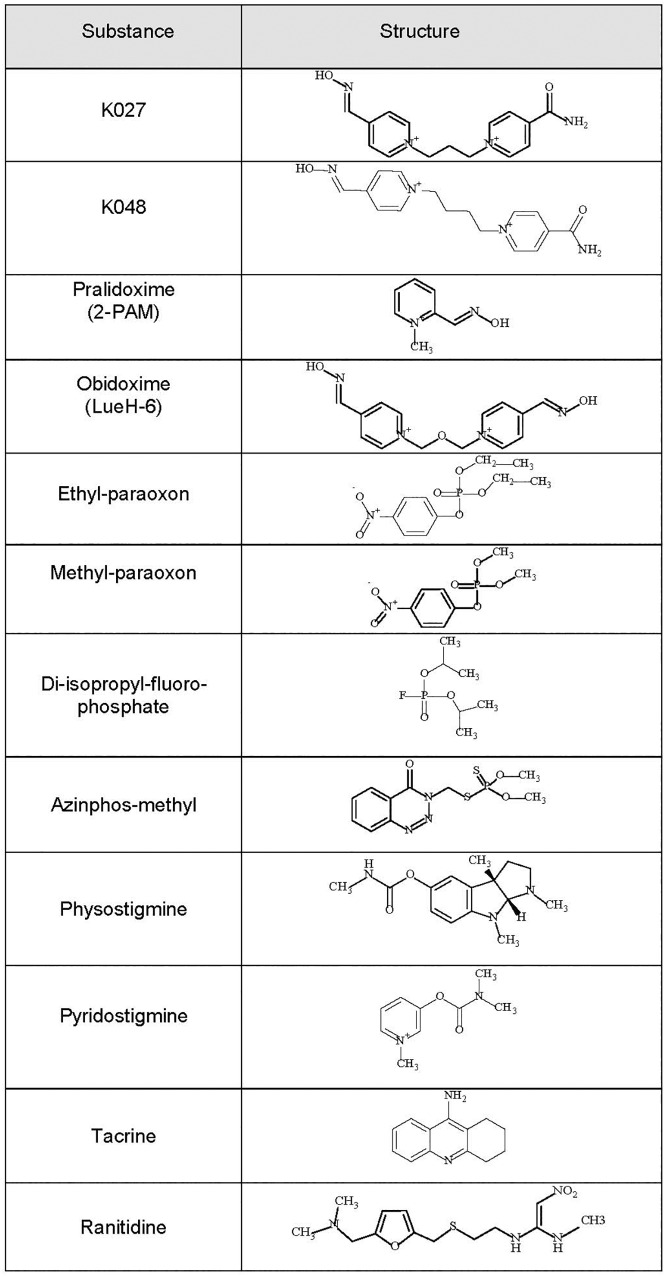
Chemical formulas of the experimental K-oximes K027 and K048, the established oximes pralidoxime and obidoxime, the organophosphorus compounds (OPCs) diisopropylfluorophosphate (DFP), ethyl-paraoxon, methyl-paraoxon, and azinphos-methyl, and the reversible acetylcholinesterase (AChE) inhibitors physostigmine, pyridostigmine, tacrine, and ranitidine.

Kamil Kuca, Kamil Musilek, and their collaborators from the Faculty of Military Health Sciences, Hradec Kralove, Czech Republic have been among the most prolific researchers, synthesizing hundreds of new oxime-type AChE reactivators, which they named K-oximes after Kamil, the first name of both Kuca and Musilek. Their first series (Kuca et al., 2003) included bisquaternary asymmetric pyridinium aldoximes containing two pyridinium rings that are connected by a propylene (**K027** = 1-(4-hydroxyiminomethyl–pyridinium)-3-(4-carbamoyl pyridinium) propane) or a butylene (**K048** = [1-(4-hydroxy iminomethyl-pyridinium)-3-(4-carbamoylpyridinium) butane]) linker, one of the pyridinium rings carrying an oxime residue in position 4, the other one a carbamoyl residue in position 4 (Figure 1). This was followed by a second series comprising bispyridinium oximes with two aldoxime groups, either in positions 2 and 4 (K053) or twice in position 4 of the pyridine rings (K074 and K075) (Musilek et al., 2007b; Kuca et al., 2009). The third series consisted of bisquaternary symmetric pyridinium aldoximes containing a xylene linker (Musilek et al., 2007a), with two functional aldoxime groups in position 2 (K106, K107, K108) or position 4 (K112, K113, K114).

Over the past 15 years, in collaboration with our Czech colleagues, we have been systematically characterizing these novel K-oximes. We have performed *in vitro* essays on human red blood cell AChE, testing the intrinsic AChE inhibitory activity of these oximes and their reactivation efficacy. In addition, we have determined their pharmacokinetic properties. These studies were followed by a series of *in vivo* experiments evaluating their efficacy to protect from OPC-induced mortality. During all these studies, the “Guiding principles in the Care of and Use of Laboratory Animals” (Council of The American Physiological Society) have been observed, and all experiments were performed with the approval of the Institutional Review Board (FMHS Animal Research Ethics Committee). We have tested these oximes, when administered immediately after the OPC diisopropylfluorophosphate (DFP), ethyl-paraoxon, methyl-paraoxon, and azinphos-methyl (Figure 1). DFP, a structural analog of the nerve agent sarin, is a widely used model compound to investigate AChE inhibition and OPC intoxications (Antonijevic and Stojiljkovic, 2007; Lorke and Petroianu, 2019). Ethyl-paraoxon = paraoxon is the biologically active metabolite of parathion, one of the earliest OPC pesticides manufactured (Konst and Plummer, 1950; Gupta, 2006b). Similarly, the pesticide methyl-parathion (metaphos), one of the most widely applied OPC pesticides, has to be bioactivated by CYP-dependent oxygenases to the very efficient AChE inhibitor methyl-paraoxon (Garcia et al., 2003; Ruckart et al., 2004; Isbister et al., 2007). Azinphos-methyl, an organophosphorothionate (thion) globally used as a broad-spectrum insecticide (Schulz, 2004; Stoner and Eitzer, 2013; Belenguer et al., 2014), which hardly inhibits AChE in its thion form, has to be metabolized *in vivo* by way of CYP450-mediated oxidative desulfuration to its highly toxic phosphate triester (oxon) form (Buratti et al., 2002). This conversion is fast, taking less than 10 min in an *in vitro* liver slice model, and 5–10 min *in vivo* after oral (Pasquet et al., 1976) or intraperitoneal (Lorke et al., 2013; Petroianu et al., 2015) administration.

Better therapeutic results are achieved when reversible AChE inhibitors are given before OPC exposure (for review, see Lorke and Petroianu, 2019). We have, therefore, also tested K027, when given as pretreatment before the same OPCs (DFP, ethyl-paraoxon, methyl-paraoxon, azinphos-methyl). Its protective efficacy was compared with that of pyridostigmine (Figure 1), the only substance approved by the US Food and Drug Administration (FDA) for pretreatment when exposure to the nerve agent soman is anticipated (US Food and Drug Administration, 2003), and of three other known AChE inhibitors (physostigmine, tacrine, ranitidine) already used clinically for other indications (reviewed in Lorke and Petroianu, 2019). Physostigmine, the first AChE inhibitor known to man, is a carbamate readily passing the blood–brain barrier that has been used in the therapy of atropine poisoning, myasthenia gravis, Alzheimer’s disease, and glaucoma (for review, see Somani and Dube, 1989; Zhao et al., 2004). The acridine derivative tacrine was the first AChE inhibitor developed to improve the cognitive performance of Alzheimer’s disease (Raina et al., 2008), and ranitidine is an inhibitor of histamine type 2 (H_2_) receptors, which is widely used to reduce gastric acid production (Grant et al., 1989).

Of the 15 evaluated K-oximes, K027 turned out to be the most promising experimental oxime. This review summarizes *in vitro* and *in vivo* results obtained for K027 and compares them with K048, the other experimental bisquaternary asymmetric pyridinium aldoximes containing two pyridinium rings, and to the most widely used established oximes pralidoxime and obidoxime.

## Pharmacokinetics

**FIGURE 2:**
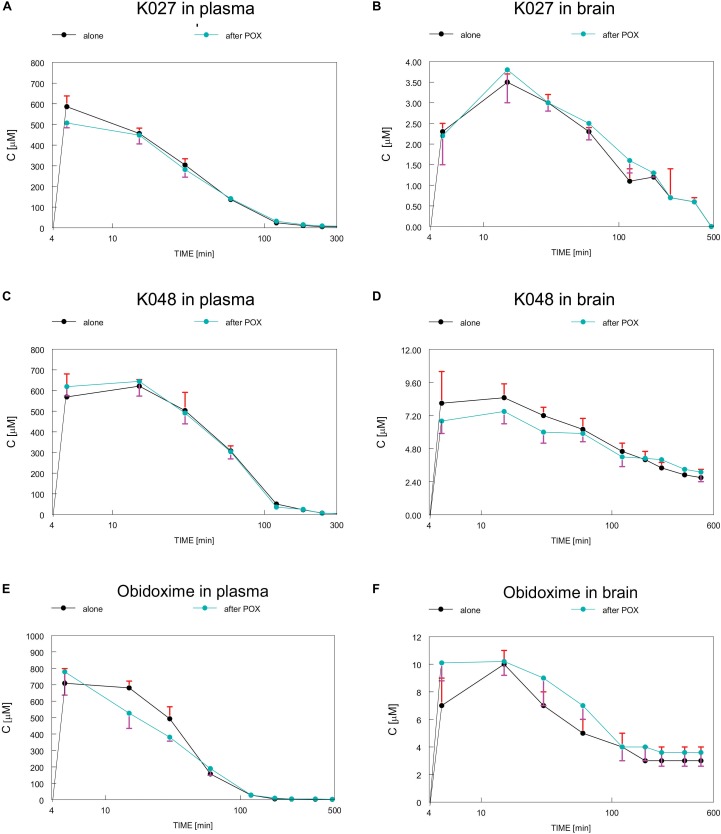
Time course of concentrations of experimental K-oximes K027 **(A,B)** and K048 **(C,D)** and the established oxime obidoxime **(E,F)** in plasma **(A,C,E)** and brain **(B,D,F)** after intramuscular (i.m.) injections of 50 μmol of oxime into rats. Depicted are concentrations, when oxime was injected alone (black) or in combination with paraoxon (POX, blue), which did not significantly influence plasma or brain concentrations of K027, K048, or obidoxime. Data from Lorke et al. (2007) and Petroianu et al. (2007b). Image reproduced with permission of “JOHN WILEY AND SONS,” License Number: 4450270092893.

Plasma and brain concentrations of K027, K048, obidoxime (Lorke et al., 2007), and pralidoxime (Petroianu et al., 2007b) were measured by high performance liquid chromatography (HPLC) (Gyenge et al., 2007) over a period of 10 h after intramuscular (i.m.) injections of 50 μmol of oxime into rats (Figure 2). Maximum plasma concentrations for pralidoxime (*C*_max_ = 303 μM), obidoxime (*C*_max_ = 716 μM), and K027 (*C*_max_ = 586 μM) were reached after 5 min (Table 1), for K048 (*C*_max_ = 621 μM) after 15 min; plasma half-life was 40 min for obidoxime and pralidoxime and 60 min for K027 and K048. In the brain, maximum concentrations were reached after 15 min for pralidoxime (*C*_max_ = 17 μM), obidoxime (*C*_max_ = 10 μM), K027 (*C*_max_ = 3.5 μM), and K048 (*C*_max_ = 8.5 μM), with a half-life of ∼70 min (pralidoxime), ∼60 min (obidoxime), ∼80 min (K027), and ∼120 min (K048). The proportion of plasma oxime entering the brain, determined as a relation between the area under the curve (AUC) plasma and the AUC brain, was 8% for pralidoxime, 5.5% for obidoxime, 2% for K027, and 5% for K048 (Table 1). Whereas a previous OPC (paraoxon) injection did not significantly affect the passage of obidoxime, K027 and K048 into the brain, brain entry of pralidoxime increased from 8% to 12% if paraoxon was administered 1 min before oxime injection (Petroianu et al., 2007b). The reasons for these differences in brain entry, for example, lipophilicity, molecular weight, and possible transporters, have been discussed in detail (Lorke et al., 2008a). Elimination kinetics of K027, when injected i.m. in the dosage of 50 μmol, have been analyzed (Tekes et al., 2006), with the possibility of zero-order kinetics (from 15 through 120 min) at high dosages.

**Table 1:** Pharmacokinetic parameters of the experimental K-oximes K024 and K048 and of the established oximes pralidoxime and obidoxime.

Oxime	*C*_max_ (Plasma)	*t*_max_ (Plasma)	*t*_1/2_ (Plasma)	*C*_max_ (Brain)	*t*_max_ (Brain)	*T*_1/2_ (brain)	Proportion (brain/plasma)	Injected dose
Pralidoxime	303 μM	5 min	40 min	17 μM	15 min	70 min	8%	50 μmol
Obidoxime	716 μM	5 min	40 min	10 μM	15 min	60 min	5.5%	50 μmol
K027	586 μM	5 min	60 min	3.5 μM	15 min	80 min	2%	50 μmol
K048	621 μM	15 min	60 min	8.5 μM	15 min	120 min	5%	50 μmol


*Column 2 lists the maximum concentration (*C*_*max*_) measured in plasma and column 3 the time after which it is reached (*t*_*max*_). Column 4 lists the half-life (*t*_*1/2*_) in plasma of the respective oxime. Columns 5–7 list the same parameters for the brain and column 8 the proportion of oxime reaching the brain, determined as the area under the concentration curve (AUC) for brain as a fraction of the AUC for plasma. Column 9 lists the dose injected intramuscularly into rats to determine pharmacokinetics. Data from Lorke et al. (2007) and Petroianu et al. (2007b).*

In a set of subsequent studies, the pharmacokinetics of K027 have been analyzed in rats after i.m. injection of a smaller oxime dosage (22.07 mg/kg = ∼12 μmol/rat). Maximum plasma concentrations (*C*_max_ = 18.6 μg/ml = 32 μM) were reached after approximately 15 min (Karasova et al., 2013) to 30 min (Karasova et al., 2013; Zemek et al., 2013), maximum brain concentrations after 15–30 min, depending on the brain region (Karasova et al., 2013), with the highest concentrations observed in the frontal cortex and the lowest in the basal ganglia. Similar observations were made for obidoxime; however, relatively low concentrations were measured in the frontal cortex for HI-6 (Karasova et al., 2014). After i.m. injections of three different K027 dosages into rats (5.0, 30, and 60 μmol/rat), maximum plasma concentrations (*C*_max_ = ∼50 mg/L = 85 μM for 60 μmol) were reached 5–15 min later, and maximum brain concentrations (*C*_max_ = ∼5 mg/L = ∼8 μM for 60 μmol) after 15–30 min (Nurulain et al., 2013), confirming overall our previous results (Lorke et al., 2007; Petroianu et al., 2007b). Binding of oximes to human serum albumin has been shown to be 5% for K027, 10% for K075, 7% for obidoxime, and 1% for HI-6 (Zemek et al., 2013). Analyzing the pharmacokinetic profile in pigs, maximum plasma concentrations were measured 20 min after i.m. injections of 1,500 mg/animal ( = 45 mg/kg = 78 μmol/kg body weight) K027 (Karasova et al., 2017), and the highest concentrations were found in the kidney and lung, whereas brain concentrations were low, the brain/plasma ratio being about 1%. K027 is highly concentrated in the urine (Karasova et al., 2017).

## *In Vitro* Parameters

After the synthesis of K027 had been described in 2003 (Kuca et al., 2003), its capacity to reactivate AChE inhibited by nerve gases has been extensively tested *in vitro* on AChE derived from rat brain homogenate. When AChE was inhibited by the nerve agent “venomous agent X” (VX) (Kuca and Kassa, 2004b), K027 displayed a relatively low reactivation potency, similar to that of obidoxime, methoxime, or pralidoxime and below that of HI-6. When the ability of K027 to reactivate AChE inhibited by the nerve agents tabun, sarin, and VX was compared with that of pralidoxime, obidoxime, and HI-6 (Kuca and Kassa, 2004a), its reactivation potency was below that of the other oximes for sarin-inhibited AChE, comparable to obidoxime for reactivation of VX- and tabun-inhibited AChE, and above HI-6 for tabun-inhibited AChE. Subsequent studies have reported efficacy of K027 and K048 to reactivate tabun-inhibited AChE (Kuca et al., 2005a), poor reactivation of sarin-inhibited AChE for K027 and K048 (Kuca et al., 2005b), and reactivation potency comparable to HI-6 for K027, when AChE was inhibited by cyclosarin (Kuca et al., 2006). Tabun-inhibited human brain AChE was best reactivated by K048, which was far superior to obidoxime and trimedoxime. K027 was only able to reactivate tabun-inhibited AChE at very high concentrations (Kuca et al., 2007). Subsequently, a comprehensive study has evaluated the potency of two different K027 concentrations to reactivate AChE derived from rat brain homogenate and inhibited by the nerve agents tabun, sarin, cyclosarin, soman, VX, or Russian VX and by the pesticides paraoxon, dicrotophos, or methylchlorpyrifos (Kuca et al., 2010). It was found that K027, at a concentration of 10 μM, could only reactivate AChE inhibited by paraoxon and methylchlorpyrifos, whereas, at a concentration of 1,000 μM, K027 also efficiently reactivated AChE inhibited by sarin, VX, Russian VX, dicrotophos, and to a much lesser degree, also tabun. Reactivation of cyclosarin- and soman-inhibited AChE by K027 was unsatisfactory. Quantum chemical, docking, and Steered Molecular Dynamics (SMD) analyses of K027 have discussed the role of its propylene linker compared with the xylene or ether linkers of other K-oximes in the reactivation of tabun-inhibited AChE (Ghosh et al., 2017). The docking results suggest that the oxime oxygen of K027 resides 5.12 Å away from the phosphorus atom of the active-site serine (SUN203) of tabun-inhibited mAChE, which is closer than K127 and K203, and the binding energy of this reactivator-protein complex is -8.20 kcal/mol.

Reactivation potencies of K027, K048, and pralidoxime have also been compared *in vitro* in human erythrocyte AChE inhibited by DFP (Lorke et al., 2008), paraoxon (Petroianu et al., 2012), and methyl-paraoxon (Petroianu et al., 2007a). In general, K027 was the most efficacious reactivator; the ranking of reactivator potencies obtained using ethyl-paraoxon as an inhibitor was as follows: K027 > K048 > K033 > pralidoxime. This ranking was basically the same as the one determined for methyl-paraoxon inhibition: K027 equals K048, which are both superior to K033, with pralidoxime being the least efficacious oxime. When erythrocyte AChE was inhibited by DFP (Lorke et al., 2008), oximes with a xylene linker (K107, K108, K113) showed better *in vitro* reactivation, whereas reactivation potency for K027, pralidoxime, and obidoxime was one order of magnitude lower. The most extensive investigation has characterized reactivation kinetics of established (pralidoxime, obidoxime, trimedoxime, HI-6, methoxime) and experimental (K027, K048, K074, K075, K108) oximes in human erythrocyte AChE inhibited by tabun, cyclosarin, and paraoxon (Winter et al., 2016). Best reactivation of paraoxon-inhibited AChE was observed for obidoxime and trimedoxime, followed by K075, K027, and K048, which were all superior to pralidoxime and HI-6. Because obidoxime, trimedoxime, K075, K027, and K048 have their oxime group in position 4, it was concluded that 4-oximes are better reactivators of paraoxon-inhibited AChE than those with the oxime group in position 2 (Winter et al., 2016). In contrast, K027, K048, obidoxime, trimedoxime, and pralidoxime were poor reactivators of cyclosarin-inhibited AChE. As a general rule, oximes with at least one oxime group in position 2 reactivate cyclosarin-inhibited AChE better than those with the oxime group in position 4 (Worek et al., 2012). Worek et al. (2012) also observed that the reactivation potency of oximes with a but-2-ene linker (K053, K075) was generally lower than the one of oximes with an oxybismethylene linker (obidoxime, HI-6), an idea that had already been put forward by Arthur Lüttringhaus and Ilse Hagedorn in the 1960s (Eyer, 2007). Only very few of the tested oximes (K074, K075, K048, trimedoxime, and to a certain degree, also K027) were able to reactivate tabun-inhibited AChE (Winter et al., 2016). Using a different model, electric eel AChE inhibited by paraoxon (Gupta et al., 2014b) and DFP (Gupta et al., 2014a), Gupta et al. have confirmed favorable reactivation kinetics of K027 and K048, which were, however, exceeded by obidoxime and trimedoxime (Gupta et al., 2014a).

Oximes are not only able to reactivate phosphylated AChE but they also themselves inhibit AChE (Lorke et al., 2008), a characteristic that is termed their “intrinsic AChE inhibitory activity.” We have quantified the intrinsic AChE inhibitory of K027 *in vitro* (Table 2) by calculating its concentration necessary to inhibit 50% of human red blood cell AChE activity (IC_50_) and compared it with that of pralidoxime, obidoxime, K048, and other experimental K-oximes (Lorke et al., 2008). The IC_50_ was measured in the presence of the selective butyrylcholinesterase inhibitor ethopropazine (Worek et al., 1999) using the method originally described by Ellman and subsequently improved by Worek (Ellman et al., 1961). Enzyme activities were corrected for oxime-induced thiocholine esteratic activity (Petroianu et al., 2004). The IC_50_ of K027 (IC_50_ = 414 μM) and K048 (IC_50 =_ 461 μM) was of the same order of magnitude as that of the established oxime pralidoxime (IC_50 =_ 592 μM) and slightly lower than the IC_50_ of obidoxime (IC_50_ = 702 μM). Weak AChE inhibition by K027 and K048 had been previously reported (Calic et al., 2006). In contrast, experimental oximes with a xylene linker (K107, K108, K113, K114) are much stronger AChE inhibitors (IC_50_ between 6 and 13 μM).

**Table 2:** Chemical and biological parameters of the experimental (K027, K048) and established (pralidoxime, obidoxime) oxime reactivators of organophosphate- inhibited acetylcholinesterase (AChE).

Oxime	Molecular weight	IC_50_ (μM)	LD_01_	LD_50_	Injected dose
					
			(μmol/rat)	(μmol/rat)	(μmol/rat)
Pralidoxime	172.6	592	117	180	50
Obidoxime	359.21	702	107	132	50
K027	446.16	414	250	350	50
K048	460.16	461	110	140	50


*Column 2 lists their molecular weights, column 3 their intrinsic ability to inhibit human erythrocyte AChE as quantified by the concentration necessary to inhibit 50% of human erythrocyte AChE activity (IC_*50*_, data from Lorke et al., 2008), column 4 their LD_*01*_, and column 5 their LD_*50*_ values (Lorke et al., 2008b) for intraperitoneal (i.p). injection in rats. Column 6 lists the doses injected i.p. within 1 min after OPC exposure in μmol/rat to test efficacy to protect from OPC-induced mortality. The injected oxime dose is approximately half the LD_01_, except for K027, which was given in a smaller dosage.*

## Toxicity

We have previously been able to show that oxime toxicity is closely related to their intrinsic AChE inhibitory activity and that the LD_50_ is correlated with the IC_50_ of AChE inhibition (Lorke and Petroianu, 2009). Correspondingly, K027 with an LD_50_ of 350 μmol/animal = 612 mg/kg body weight (i.p. injection) is the least toxic of the tested oximes in rats (Table 2), compared with 180 μmol/animal = 120 mg/kg body weight for pralidoxime, 140 μmol/animal = 246 mg/kg body weight for K048, and 132 μmol/animal = 177 mg/kg body weight for obidoxime, which is more than one order of magnitude less lethal than the oximes with a xylene linker (LD_50_ = ∼3–15 μmol/animal) (Lorke and Petroianu, 2009). Almost identical LD_50_ values for K027 and K048 were reported by Berend et al. (2008), and LD_50_ figures obtained for mice also came very close to our data (Kassa et al., 2007). Slightly higher values were obtained after i.m. injections (Antonijevic et al., 2016): 2.53 mmol/kg for K027, compared with ∼1.4 mmol/kg for i.p. injection; 0.49 mmol/kg both for i.m. and i.p. administration of obidoxime and 1.24 mmol/kg for pralidoxime i.m. versus ∼0.7 mmol/kg for i.p. injection. In addition, it needs to be taken into account that the IC_50_ of K027, determined in rat blood, is two and one half times higher than the one determined in human blood (Lorke and Petroianu, 2009), which may indicate higher toxicity in humans than in rats.

In comparison to oximes, OPCs are much more potent AChE inhibitors (Table 3). AChE inhibition of the tested OPCs, with the exception of azinphos-methyl, is about three orders of magnitude more potent than the intrinsic AChE inhibitory activity of the investigated oximes (for review, see Lorke and Petroianu, 2019), with an IC_50_ of 15 nM for ethyl-paraoxon, 60 nM for methyl-paraoxon, and 120 nM for DFP. The IC_50_ of azinphos-methyl (189 μM) is relatively high in comparison, but it has to be kept in mind that azinphos-methyl is a thiophosphate that needs to be bioactivated in the liver to its oxon form to become a potent AChE inhibitor. Correspondingly, all four OPCs have an LD_50_ in the 1- to 6-μmol/animal range (Table 3).

**Table 3:** Chemical and biological parameters of the investigated OPCs.

Organophosphorus compound	Molecular weight	IC_50_ (μM)	LD_50_ (μmol/rat)	LD_70_ (μmol/rat)	Injected dose (μmol/rat)
DFP	184.15	0.12	4.8	6	6, 10, 14
Ethyl-paraoxon	275.2	0.015	0.8	1	1, 5, 10
Methyl-paraoxon	247.14	0.06	1.1	1.5	2, 3, 5
Azinphos methyl	317.32	189	3.2	5	5, 10, 15


*Column 2 lists their molecular weights, column 3 their concentration necessary to inhibit 50% of human red blood cell AChE activity (IC_*50*_), column 4 their LD_*50*_ values for intraperitoneal (i.p). application in rats, column 5 their LD_*70*_ values, and column 6 the doses injected i.p. to perform a Cox (1972) analysis of the mortality-reducing efficacy of various oximes, including K027. The administered doses are approximately LD_*70*_, LD_*100*_, and twice LD_*70*_.*

Several *in vitro* studies have been performed assessing the toxicity of K027. In addition to measuring the intrinsic AChE inhibitory activity and LD_50_ values (see above), the interaction of K027, K075 and other experimental oximes with calf thymus DNA has been tested, and changes in cell cycle distribution, mitochondrial membrane potential, and cell viability have been determined in HL-60 (human acute promyelocytic leukemia) cells (Janockova et al., 2014). In that study, K027 and K075 were found to be relatively safe from the point of view of DNA binding, and there was no indication for cytotoxicity. Because hepatotoxicity has been reported for several of the established oximes (for review see Marrs, 1991), the influence of K027 on cell viability has been tested in hepatocellular cell lines. No prominent inhibition of the activities of human liver microsomal cytochromes P450 by K027 was detected (Spicakova et al., 2016), and there was no indication that K027 or pralidoxime impaired the viability of human hepatocellular carcinoma (HepG2) cells (Prado et al., 2015). K027 and pralidoxime had no effect on glycolysis or oxygen consumption in HepG2 cells (Prado et al., 2015). Moreover, these two oximes did not lead to the generation of oxidants nor did they affect the mitochondrial membrane potential. In addition, K027 and pralidoxime failed to activate effector caspases (Prado et al., 2015). The same parameters were not altered in human neuroblastoma (SH-SY5Y) cells either, and there was no indication for neurotoxicity other than increased ACh levels due to AChE inhibition (Prado et al., 2015). Another study, examining the influence of three K- (K027, K074, K075) and five established oximes (pralidoxime, trimedoxime, obidoxime, methoxime, HI-6) on the viability of the same hepatocellular carcinoma cells (HepG2), did not give any indication for hepatotoxicity either (Muckova et al., 2019). In the MTT (dimethylthiazol-diphenyl-tetrazolium bromide) reduction assay, evaluating mitochondrial succinate dehydrogenase activity, and the “electrical impedance based real-time cytotoxicity assay” (Muckova et al., 2019), K027 was the least cytotoxic oxime for hepatocyte and fibroblast cell lines and the second least toxic oxime for adenocarcinoma cell lines. Taken together, there is no *in vitro* indication that K027 is hepatotoxic or neurotoxic, apart from its intrinsic cholinesterase inhibitory activity due to its interaction with the catalytic site of the AChE enzyme (Lorke et al., 2008).

*In vivo* studies on rats (Lorke et al., 2008) and mice (Zunec et al., 2015) also demonstrate that K027 and K048 stand out by their low acute toxicities. Compared with established and other K-oximes, K027 is the least toxic, with regard to its LD_50_ (see above). Histopathological evaluation of the effects of K027, HI-6, and obidoxime on hepatic functions in rats *in vivo* showed no influence of these oximes on the number of lipid droplets in liver tissue samples, but a decrease in multidrug resistance protein 2 immunoreaction after injection of HI-6 at a dosage of 5% LD_50_ (Pejchal et al., 2008). A comparable effect was only achieved after the injection of 50% LD_50_ of K027 and obidoxime. Whereas lower doses of HI-6 and obidoxime were able to impair hepatic transporter function, K027 only affected the transporter at much higher concentrations (Pejchal et al., 2008).

## *In Vivo* Protection

The efficacy of K027 to protect from tabun-induced toxicity has been demonstrated in a number of *in vivo* experiments. The efficacy of K027 and K048 to reactivate *in vivo* AChE in rat blood, diaphragm, and brain tissue has been compared with that of obidoxime, trimedoxime, and HI-6 after i.m. injections of tabun (Kassa et al., 2006). AChE measurements have revealed that the *in vivo* reactivating efficacy of K027 and K048 is comparable to the efficacy of obidoxime and trimedoxime and that K027 and K048 can also eliminate the acute lethal effects of tabun (Kassa et al., 2006). Monitoring a large battery of behavioral changes following administration of sublethal tabun doses to rats, it was demonstrated that tabun-induced neurotoxicity could be reduced, but not completely eliminated by K027, K048, and obidoxime in combination with atropine, whereas HI-6 was not efficacious (Kassa and Kunesova, 2006a). Similarly, the cognitive performance after tabun exposure could be improved by K027 plus atropine (Kassa and Kunesova, 2006b). In addition, K027 and K048 better protected mice from tabun-induced mortality than HI-6, whereas their mortality-reducing efficacy on soman toxicity was inferior to that of HI-6 (Calic et al., 2006; Berend et al., 2008). When combined with the anticholinergic benactyzine, K027 and K048 were as efficacious as trimedoxime and superior to pralidoxime, obidoxime, and HI-6 in protecting mice from acute tabun-induced mortality (Kassa, 2006). Overall, with regard to nerve agents, K027 was efficacious *in vivo* to protect from the toxic effects of tabun, but results for cyclosarin and soman were unsatisfactory (Kuca et al., 2010; Antonijevic et al., 2016).

When tested in rats exposed to the OPC pesticide dichlorvos, K027 was more efficacious in reducing the dichlorvos-induced lethal effects than the established oximes pralidoxime, trimedoxime, obidoxime, and HI-6, when given immediately after OPC administration (Antonijevic et al., 2016) and reactivated dichlorvos-inhibited AChE *in vivo* more efficiently than K203 (Antonijevic et al., 2018b). Compared with pralidoxime, obidoxime, trimedoxime, and HI-6, K027 also best reduced oxidative stress induced by dichlorvos poisoning (Antonijevic et al., 2018a).

In a standardized experimental setting (shown in Tables 2, 3), we have quantified *in vivo* the protection conferred by K027 to reduce mortality induced by DFP and the pesticides ethyl-paraoxon, methyl-paraoxon, and azinphos-methyl and compared it with established (pralidoxime, obidoxime, trimedoxime, HI-6) and experimental (K048, K053, K074, K075, K107, K108, K113) oximes (Lorke et al., 2008b; Nurulain et al., 2009; Petroianu et al., 2012; Lorke et al., 2013). The relative risk (RR) of death over time was calculated according to Cox survival analysis (Cox, 1972) in rats that had been administered one of the OPCs at three dosages (∼LD_70_, ∼LD_100_, ∼2 × LD_70_; Table 3) and one of the oximes in a biologically defined dosage (50% of LD_01_) within 1 min thereafter (Table 2). Cox regression model allows for a statistical analysis of two different survival curves. Instead of only looking at the survival at one predetermined time point, the comparison of two survival curves measured over an extended period provides additional information. Moreover, the Cox proportional hazards model also allows for the analysis of several factors of known or likely importance for the survival of the animals (Gill, 1982). In our case, these covariates have been OPC dose and type of treatment. Mortality data have been compared and, for each of the seven time points, the respective hazard ratios (relative risks of death) have been estimated using the Cox proportional hazards model (Cox, 1972). Both OPC dose (LD_100_, 2 × LD_70_, respectively, with LD_70_ the reference category) and type of treatment (with the no treatment group as the reference category) have been considered as categorical variables. The RR equals 1 in animals that have only received the OPC but no oxime treatment. A lower RR signifies superior oxime protective efficacy.

K027 was the most efficacious protector from DFP-induced toxicity, reducing the RR to 0.16 (Table 4), which was significantly better than pralidoxime (RR = 0.62). Obidoxime (RR = 0.19) and K048 (RR = 0.28) also significantly reduced DFP-induced mortality (Lorke et al., 2008b). In animals exposed to ethyl-paraoxon (Nurulain et al., 2009), best efficacy was again observed for K027 (RR = 0.20), which was significantly better than any other tested oxime. Significant reduction was also observed for K048 (RR = 0.32), obidoxime (RR = 0.64), and pralidoxime (RR = 0.78). K027 (RR = 0.58) and K048 (RR = 0.60) were the only oximes investigated that were able to significantly reduce methyl-paraoxon-induced mortality (Table 4); none of the established oximes (pralidoxime, obidoxime, trimedoxime, and HI-6) was efficacious against methyl-paraoxon (Petroianu et al., 2012). When animals were exposed to azinphos-methyl (Lorke et al., 2013), K027 also significantly reduced the relative risk of death (RR = 0.26). A slightly, but not significantly, better protection from the lethal effects of azinphos-methyl was observed for K053 (RR = 0.22). Significant reduction from azinphos-methyl-induced mortality was also achieved by K048 (RR = 0.33), obidoxime (RR = 0.37), and pralidoxime (RR = 0.39). In summary, when given immediately after exposure to the OPCs DFP, ethyl-paraoxon, methyl-paraoxon, dichlorvos, and azinphos-methyl, K027 is, by far, the most efficacious of the tested oximes.

**Table 4:** Protective efficacy of the tested oximes, assessed by Cox analysis (Cox, 1972) of the cumulative relative risk (RR) of death of animals exposed to the OPCs diisopropylfluorophosphate = DFP, ethyl-paraoxon = paraoxon, azinphos-methyl or methyl-paraoxon and, within 1 min thereafter, treated with the experimental K-oximes (K027, K048) or the established oximes (pralidoxime, obidoxime).

Organophosphorus compounds		DFP^1^	Ethyl-paraoxon^2^	Azinphos-methyl^3^	Methyl-paraoxon^4^
Oxime	Pralidoxime	**0.62 ± 0.09**	**0.78 ± 0.06**	**0.39 ± 0.10**	0.88 ± 0.10
	Obidoxime	**0.19 ± 0.07**	**0.64 ± 0.14**	**0.37 ± 0.10**	0.93 ± 0.10
	K027	**0.16 ± 0.06**	**0.2 ± 0.06**	**0.26 ± 0.11**	**0.58 ± 0.14**
	K048	**0.28 ± 0.06**	**0.32 ± 0.08**	**0.33 ± 0.07**	**0.6 ± 0.15**


*The cumulative RR was assessed by determining the area under the RR-time curve, adjusted for OPC dose (high/low). RR = 1 for the reference group (OPC alone, no pretreatment); lower values reflect better protection. As illustrated by this table, by far the best RR-reducing efficacy is achieved by K027. ^*1*^Data from Lorke et al. (2008b), ^*2*^data from Lorke and Petroianu (2009) and Nurulain et al. (2009), ^*3*^data from Lorke et al. (2013), and ^*4*^data from Petroianu et al. (2012). Bold Values: Oxime treatment significantly (*p* ≤ 0.05) decreased the RR of OPC-induced death. Statistical differences were tested by the Mann–Whitney *U* test and a *p*-value ≤ 0.05 was considered significant.*

In addition, we have tested whether K027 is also able to protect from OPC-induced toxicity if administered before OPC exposure. Such pre-exposure treatment has been utilized in the 1991 Gulf War (Keeler et al., 1991; McCauley, 2006; Pope, 2006); and thereafter, the US FDA has given its approval to administer pyridostigmine orally, when soman exposure is imminent (US Food and Drug Administration, 2003). Pretreatment with pyridostigmine is, however, only effective when atropine and oxime are given after OPC exposure (Tuovinen et al., 1999; Masson and Nachon, 2017), and frequent, although not incapacitating side effects have been reported (McCauley, 2006; Masson and Nachon, 2017). In contrast, physostigmine, which is able to cross the blood–brain barrier, administered together with the anticholinergic drug scopolamine, is more efficacious prophylactically than pyridostigmine (Wetherell et al., 2007). However, unwanted behavioral effects, which may affect decision-making and adequate reactions in critical situations, have been described as well (Masson and Nachon, 2017).

In search of an effective, practicable, acceptable, and affordable pretreatment substance, we have evaluated *in vivo* the prophylactic efficacy of a number of reversible cholinesterase inhibitors (physostigmine, pyridostigmine, tacrine, ranitidine, K027) by calculating the RR over time (Cox, 1972) in rats that were first given the prophylactic agent at an equitoxic dosage (25% of LD_01_; Table 5) and thereafter (30 min later) one of the OPCs at three equitoxic dosages (∼LD_70_, ∼LD_100_, ∼2 × LD_70_; Table 3). When comparing the efficacy of different prophylactic agents, they have to be administered in comparable dosages. We decided to administer quantities according to *in vivo* toxicity, that is, 25% of LD_01_ (= 25% of the dose at which 1% of the animals die). This is an amount well tolerated by the experimental animals (Lorke et al., 2011). We have previously elaborated why equidosing according to *in vitro* parameters, for example, IC_50_ of AChE inhibition, would ignore toxicities unrelated to AChE inhibition and therefore produce false-negative results (Lorke et al., 2011; Petroianu et al., 2013; Lorke and Petroianu, 2019).

**Table 5:** Chemical and biological parameters of the investigated inhibitors of AChE administered prophylactically before exposure to OPCs.

Reversible AChE Inhibitor	Molecular Weight	IC_50_ (μM)	LD_01_ (μmol/rat)	LD_50_ (μmol/rat)	Injected Dose (μmol/rat)
Physostigmine	275.35	0.012	0.9	3.0	0.25
Pyridostigmine	261.12	0.33	3.7	7.2	1
Tacrine	250.00	0.2	16	21.5	4.00
Ranitidine	350.86	2.5	46	59	12.00
K027	446.16	414	250	350	60.00


*Column 2 lists their molecular weights, column 3 their concentration necessary to inhibit 50% of human red blood cell AChE activity (IC_*50*_) (Lorke et al., 2011), column 4, their LD_*50*_ values for intraperitoneal (i.p.) application in rats, column 5 their LD_*01*_ values, and column 6 the doses injected i.p. for pretreatment 30 min before OPC exposure to assess their efficacy to protect from OPC-induced mortality. The injected doses are approximately one fourth the LD_*01*_.*

Such pretreatment is only feasible in situations when OPC exposure can be anticipated, be it due to chemical warfare, terrorist attacks, or exposure of rescue personal to contaminated individuals. Given the diversity of OPCs that can possibly be used for malicious purposes, ranging from an improvised bomb containing pesticides fabricated by Hamas in 1997 (Dolnik and Bhattacharjee, 2002; Kostadinov et al., 2010) to the recent sarin gas attacks in the Syrian war (Dolgin, 2013; Pita and Domingo, 2014), potential prophylactic agents need to be tested against a broad range of chemically diverse OPCs. The prophylactic efficacy of the individual pretreatment compounds tested in our experiments varied depending on the OPCs administered (Table 6), as previously reported for nerve agents (Tuovinen et al., 1999).

**Table 6:** Protective efficacy of K027 and reversible AChE inhibitors (physostigmine, pyridostigmine, tacrine, ranitidine) administered prophylactically before exposure to the OPCs ethyl-paraoxon = paraoxon, methyl-paraoxon or azinphos-methyl.

Organophosphorus compounds		DFP^1^	Ethyl-paraoxon^2^	Methyl-paraoxon^3^	Azinphos-methyl^4^
Reversible AChE Inhibitors	Physostigmine	**0.02 ± 0.01**	**0.3 ± 0.15**	**0.39 ± 0.08**	**0.21 ± 0.5**
	Pyridostigmine	**0.28 ± 0.09**	**0.76 ± 0.13**	0.98 ± 0.11	**0.37 ± 0.11**
	Tacrine	**0.05 ± 0.03**	**0.67 ± 0.21**	**0.48 ± 0.07**	**0.29 ± 0.06**
	Ranitidine	**0.41 ± 0.07**	**0.72 ± 0.16**	0.87 ± 0.17	**0.62 ± 0.29**
	K027	**0.18 ± 0.12**	**0.34 ± 0.09**	**0.4 ± 0.13**	**0.15 ± 0.09**


*The reduction in the relative risk of death (RR) is calculated according to Cox (1972), with an RR = 1 for the reference group (OPC alone, no pretreatment). Lower values reflect better protection. As illustrated by this table, the best RR-reducing efficacies were observed for K027 and physostigmine. ^*1*^Data from Lorke et al. (2011), ^*2*^data from Petroianu et al. (2013), ^*3*^data from Lorke et al. (2012), and ^*4*^data from Petroianu et al. (2015). Bold values: Pretreatment significantly (*p* ≤ 0.05) decreased the RR.*

Optimal protection from DFP-induced mortality (Lorke et al., 2011) was afforded by physostigmine (RR = 0.02) and tacrine (RR = 0.05), which were significantly (*p* ≤ 0.05) more efficacious than any of the other tested AChE inhibitors, except K027 (RR = 0.18). Protection afforded by pyridostigmine (RR = 0.28) and ranitidine (RR = 0.41) was also statistically significant, but inferior to physostigmine and tacrine. When given before ethyl-paraoxon exposure (Petroianu et al., 2013), best protection was achieved by pretreatment with physostigmine (RR = 0.30) and K027 (RR = 0.34), which were significantly more efficacious than the other tested substances. Prophylactic administration of tacrine (RR = 0.67), ranitidine (RR = 0.72), and pyridostigmine (RR = 0.76) also significantly reduced ethyl-paraoxon-induced mortality as compared with the non-treatment group (paraoxon only, RR = 1; Table 6). In the case of methyl-paraoxon exposure (Lorke et al., 2012), only pretreatment with physostigmine (RR = 0.39), K027 (RR = 0.40), and tacrine (RR = 0.48) significantly reduced mortality; pyridostigmine and ranitidine did not significantly improve the RR. Mortality due to azinphos-methyl (Petroianu et al., 2015) was best prevented by K027 (RR = 0.15) and physostigmine (RR = 0.21), followed by tacrine (RR = 0.29), pyridostigmine (RR = 0.37), and ranitidine (RR = 0.62), the latter being significantly less efficacious than physostigmine, tacrine, and K027.

In summary, K027, when given prophylactically, very efficaciously protects from exposure to a variety of OPCs, also including terbufos sulfone (Lorke et al., 2014) and dicrotophos (Lorke et al., 2017). We hypothesize that it acts by reactivating phosphylated AChE rather than protecting the enzyme from phosphorylation (Lorke and Petroianu, 2019).

## Conclusion

K027 *in vitro* efficiently reactivates cholinesterase inhibited by a broad range of organophosphates. It achieves maximum plasma concentrations shortly after i.m. injection; and only a negligible percentage is able to cross the blood–brain barrier. Due to its low toxicity, K027 can be given in high dosages, which makes it a very promising oxime not only for post-exposure treatment but also for prophylactic administration, especially when brain penetration is undesirable. Although K027 protects against a broad range of pesticides and several nerve agents, it is not efficacious against all nerve agents. Further improvement may be achieved by combining K027 with other low-toxicity oximes with a complementary spectrum (Worek et al., 2016).

## Author Contributions

DL wrote the manuscript. GP reviewed the manuscript and added significant parts.

## Conflict of Interest Statement

The authors declare that the research was conducted in the absence of any commercial or financial relationships that could be construed as a potential conflict of interest. The handling Editor declared a past co-authorship with one of the authors GP.
